# Supplementary material for: Circuit mechanisms encoding odors and driving aging-associated behavioral declines in Caenorhabditis elegans
Source: eLife. 2015 Sep 22;4:e10181. doi: 10.7554/eLife.10181 (PMC4577979; doi:10.7554/eLife.10181)
Supplement: Supplementary file 1. — C. elegans strain list. DOI: http://dx.doi.org/10.7554/eLife.10181.043 [file elife10181s020.pdf]

Supplementary file 1. *C. elegans* strain list.

Strain	Genotype	Name and Figures
**Calcium imaging: wild-type GCaMP strains**		
CX10536	*kyEx2595 [str-2::GCaMP2.2b, unc-122::gfp]*	"AWC^{ON}" in Figures 1,5 and Figure 1–figure supplement 1, Figure 5–figure supplements 1-4. "WT" in Figure 3A and Figure 4–figure supplement 1A-C. Also used for all conditions in Figure 2A.
PS6253	*pha-1(e2123) III; syEx1238 [srsx-3::GCaMP3, pha-1::pha-1]*	"AWC^{OFF}" in Figure 1, Figure 1–figure supplement 1. "WT" in Figure 3–figure supplement 1A
IV388	*ueEx7 [gcy-7::GCaMP3, unc-122::gfp]*	"ASEL" in Figures 1,5 and Figure 1–figure supplement 1, Figure 5–figure supplements 1-4. "WT" in Figures 3C, 4A-D and 6B,F and Figure 3–figure supplement 1C, Figure 5–figure supplement 5A,E and Figure 6–figure supplement 1A-C. Also used for all conditions in Figure 2C and Figure 5–figure supplement 5C,G.
IV28	*ueEx10 [gcy-5::GCaMP3, unc-122::gfp]*	"ASER" in Figure 1, Figure 1–figure supplement 1. "WT" in Figure 3–figure supplement 1B
PY6554	*oyEx [gpa-4::GCaMP2.2b, unc-122::dsRed]*	"AWA" in Figures 1 and 5 and Figure 1–figure supplement 1, Figure 5–figure supplements 1,3. "WT" in Figure 3B and Figure 4–figure supplement 1E,F. "ASI" in Figure 1 and Figure 1–figure supplement 1. Also used for all conditions in Figure 2B.
PY7336	*oyEx [str-1::GCaMP3, unc-122::dsRed]*	"AWB" in Figures 1 and 5 and Figure 1–figure supplement 1, Figure 5–figure supplements 1-4. "WT" in Figures 3D,4E,4F,7B,7E and Figure 3–figure supplement 1D, Figure 4–figure supplement 1D, Figure 5–figure supplement 5B,F and Figure 7–figure supplement1A-C. Also used for all conditions in Figure 2D and Figure 5–figure supplement 5D,H.

IV346	*kyEx2865 [sra-6::GCaMP3, unc-122::gfp]*	"ASH" in Figure 1, Figure 1–figure supplement 1, Figure 5–figure supplement 3
CX10981	*kyEx2866 [sra-9::GCaMP2.2b, unc-122::gfp]*	"ASK" in Figure 1, Figure 1–figure supplement 1
IV413	*ueEx254 [srh-142::GCaMP3, unc-122::gfp]*	"ADF" in Figure 1, Figure 1–figure supplement 1
CX12022	*kyEx3290 [sre-1:: GCaMP3, unc-122::dsRed]*	"ADL" in Figure 1, Figure 1–figure supplement 1
IV69	*oyEx [gcy-8::GCaMP3, unc-122::rfp]*	"AFD" in Figure 1, Figure 1–figure supplement 1
OF977	*ixEx196 [ops-1::GCaMP3, lin-44::rfp]*	"ASG" in Figure 1, Figure 1–figure supplement 1
ZD1184	*qdEx103 [trx-1::GCaMP5, unc-122::gfp]*	"ASJ" in Figure 1, Figure 1–figure supplement 1
**Calcium imaging: mutant, rescue and overexpression GCaMP strains**		
IV15	*unc-13(e51) I; kyEx2595 [str-2::GCaMP2.2b, unc-122::gfp]*	"*unc-13*" in Figure 3A
IV23	*unc-31(e928) IV; kyEx2595 [str-2::GCaMP2.2b, unc-122::gfp]*	"*unc-31*" in Figure 3A
IV141	*unc-13(e51) I; pha-1 (e2123) III; syEx1238 [srsx-3::GCaMP3, pha-1::pha-1]*	"*unc-13*" in Figure 3–figure supplement 1A
IV44	*unc-31(e928) IV; pha-1 (e2123) III; syEx1238 [srsx-3::GCaMP3, pha-1::pha-1]*	"*unc-31*" in Figure 3–figure supplement 1A
IV244	*unc-13(e51) I; oyEx [gpa-4::GCaMP2.2b, unc-122::dsRed]*	"*unc-13*" in Figure 3B
IV234	*unc-31(e928) IV; oyEx [gpa-4::GCaMP2.2b, unc-122::dsRed]*	"*unc-31*" in Figure 3B
IV104	*unc-13(e51) I; ueEx7 [gcy-7::GCaMP3, unc-122::gfp]*	"*unc-13*" in Figure 3–figure supplement 1C
IV22	*unc-31(e928) IV; ueEx7 [gcy-7::GCaMP3, unc-122::gfp]*	"*unc-31*" in Figure 3C
IV54	*unc-31(e928) IV; ueEx7 [gcy-7::GCaMP3, unc-122::gfp]; kyEx875 [odr-3::unc-31, elt-2::gfp]*	"*unc-31; AWC::unc-31*" in Figure 3C
IV68	*unc-13(e51) I; ueEx10 [gcy-5::GCaMP3, unc-122::gfp]*	"*unc-13*" in Figure 3–figure supplement 1B
IV32	*unc-31(e928) IV; ueEx10 [gcy-5::GCaMP3, unc-122::gfp]*	"*unc-31*" in Figure 3–figure supplement 1B
IV383	*unc-13(e51) I; oyEx [str-1::GCaMP3, unc-122::dsRed]*	"*unc-13*" in Figure 3D
IV381	*unc-31(e928) IV; oyEx [str-1::GCaMP3, unc-122::dsRed]*	"*unc-31*" in Figure 3–figure supplement 1D
IV193	*ins-1(nr2091) IV; ueEx7 [gcy-7::GCaMP3,*	"*ins-1*" in Figure 4A

	unc-122::gfp]	
IV245	*ins-1(nr2091) IV; ueEx7 [gcy-7::GCaMP3, unc-122::gfp]; ueEx152 [odr-3::ins-1:sl2mCherry, unc-122::rfp]*	"*ins-1; AWC::ins-1*" in Figure 4A
IV634	*ins-1(nr2091) IV; ueEx7 [gcy-7::GCaMP3, unc-122::gfp]; ueEx428 [gpa-4deletion::ins-1:sl2mCherry, unc-122::rfp]*	"*ins-1; AWA::ins-1*" in Figure 4A
IV655	*ueEx7 [gcy-7::GCaMP3, unc-122::gfp]; ueEx448 [odr-3::ins-1 sense:sl2mCherry, odr-3::ins-1 antisense:sl2mCherry, unc-122::rfp]*	*"AWC::ins-1 RNAi"* in Figure 4B,G
CX10926	*ins-1(nr2091) IV; kyEx2595 [str-2::GCaMP2.2b, unc-122::gfp]*	"*ins-1*" in Figure 4–figure supplement 1A
IV172	*daf-2(e1370) III; ueEx7 [gcy-7::GCaMP3, unc-122::gfp]*	"*daf-2*" in Figure 4C
IV224	*daf-2(e1370) III; ueEx7 [gcy-7::GCaMP3, unc-122::gfp]; ueEx139 [gcy-7::daf-2:sl2mCherry, unc-122::rfp]*	"*daf-2; ASEL::daf-2*" in Figure 4C
IV175	*daf-2(e1370) III; kyEx2595 [str-2::GCaMP2.2b, unc-122::gfp]*	"*daf-2*" in Figure 4–figure supplement 1B
IV96	*age-1(hx546) II; ueEx7 [gcy-7::GCaMP3, unc-122::gfp]*	"*age-1*" in Figure 4D
IV114	*age-1(hx546) II; ueEx7 [gcy-7::GCaMP3, unc-122::gfp]; ueEx54 [gcy-7::age-1:sl2mCherry, unc-122::rfp]*	"*age-1; ASEL::age-1*" in Figure 4D
IV98	*age-1(hx546) II; kyEx2595 [str-2::GCaMP2.2b, unc-122::gfp]*	"*age-1*" in Figure 4–figure supplement 1C
IV401	*unc-17(e245) IV; oyEx [str-1::GCaMP3, unc-122::dsRed]*	"*unc-17*" in Figure 4E, Figure 4–figure supplement 1F
IV487	*unc-17(e245) IV; oyEx [str-1::GCaMP3, unc-122::dsRed]; ueEx305 [gpa-4deletion::unc-17:sl2mCherry, unc-122::gfp]*	"*unc-17; AWA::unc-17*" in Figure 4E
IV539	*unc-17(e245) IV; oyEx [gpa-4::GCaMP2.2b, unc-122::dsRed]*	"*unc-17*" in Figure 4–figure supplement 1D
IV416	*oyEx [str-1::GCaMP3, unc-122::dsRed]; ueEx257 [gpa-4deletion::TeTX:sl2mCherry, unc-122::gfp]*	*"AWA::TeTX"* in Figure 3D
IV392	*oyEx [str-1::GCaMP3, unc-122::dsRed]; ueEx131 [odr-3::TeTX:sl2mCherry, elt-2::gfp]*	*"AWC::TeTX"* in Figure 3D
IV216	*ueEx131 [odr-3::TeTX:sl2mCherry, elt-2::gfp]*	*"AWC::TeTX"* in Figure 8–figure supplement 2B
IV640	*oyEx [str-1::GCaMP3, unc-122::dsRed]; ueEx434[gpa-4deletion::cho-1*	*"AWA::cho-1 RNAi"* in Figure 4F

	sense:sl2mCherry, gpa-4deletion::cho-1 antisense:sl2mCherry, unc-122::gfp]	
IV644	*oyEx [str-1::GCaMP3, unc-122::dsRed]; ueEx438[gpa-4deletion::cha-1 sense:sl2mCherry, gpa-4deletion::cha-1 antisense:sl2mCherry, unc-122::gfp]*	*"AWA::cha-1 RNAi"* in Figure 4F,H
IV654	*oyEx [gpa-4::GCaMP2.2b, unc-122::dsRed]; ueEx438[gpa-4deletion::cha-1 sense:sl2mCherry, gpa-4deletion::cha-1 antisense:sl2mCherry, unc-122::gfp]*	*"AWA::cha-1 RNAi"* in Figure 4–figure supplement 1E
IV469	*glp-1(e2141ts) III; ueEx7 [gcy-7::GCaMP3, unc-122::gfp]*	*"glp-1"* in Figure 5–figure supplement 5A,E
IV461	*glp-1(e2141ts) III; oyEx [str-1::GCaMP3, unc-122::dsRed]*	*"glp-1"* in Figure 5–figure supplement 5B,F
IV532	*ueEx7 [gcy-7::GCaMP3, unc-122::gfp]; uthIs202 [aak-2::aak-2(aa1-aa321 gf):Tomato, rol-6]*	*"aak-2(gf)"* in Figure 5–figure supplement 5C,G
IV537	*oyEx [str-1::GCaMP3, unc-122::dsRed]; uthIs202 [aak-2::aak-2(aa1-aa321 gf):Tomato, rol-6]*	*"aak-2(gf)"* in Figure 5–figure supplement 5D,H
IV404	*ueEx7 [gcy-7::GCaMP3, unc-122::gfp]; ueEx247 [ceh-36deletion::tom-1 sense:sl2mCherry, ceh-36deletion::tom-1 antisense:sl2mCherry, unc-122::rfp]*	*"AWC::tom-1 RNAi"* in Figures 6,8E and Figure 6–figure supplement 1
IV467	*ueEx7 [gcy-7::GCaMP3, unc-122::gfp]; ueEx292 [odr-3::ins-1(OE):sl2mCherry, unc-122::rfp]*	*"AWC::ins-1(OE)"* in Figures 6,8E and Figure 6–figure supplement 1
IV511	*ueEx7 [gcy-7::GCaMP3, unc-122::gfp]; ueEx321 [gcy-7::daf-2(OE):sl2mCherry, unc-122::rfp]*	*"ASEL::daf-2(OE)"* in Figure 6 and Figure 6–figure supplement 1
IV497	*oyEx [str-1::GCaMP3, unc-122::dsRed]; ueEx310 [gpa-4deletion::unc-17(OE):sl2mCherry, unc-122::gfp]*	*"AWA::unc-17(OE)"* in Figures 7,8D and Figure 7–figure supplement 1
IV596	*che-1(p672) I; ueEx247 [ceh-36deletion::tom-1 sense:sl2mCherry, ceh-36deletion::tom-1 antisense:sl2mCherry, unc-122::rfp]*	*"ASE(-); AWC::tom-1 RNAi"* in Figure 6G
IV598	*peIs1715 [str-1::mCasp-1, unc-122::mCherry]; ueEx310 [gpa-4deletion::unc-17(OE):sl2mCherry, unc-122::gfp]*	*"AWB(-); AWA::unc-17(OE)"* in Figure 7F
**Additional strains for behavior**		
N2	Bristol strain	"WT" in all behavior and lifespan figures
PY7502	*oyIs [ceh-36del::caspase-3(p12)::nz, ceh-36del::cz::caspase-3(p17), srtx-1::gfp,*	"AWC(-)" in Figures 1E,5J

	unc-122::dsRed]	
IV658	*ueEx257 [gpa-4deletion::TeTX:sl2mCherry, unc-122::gfp]*	*"AWA::TeTX"* in Figures 1E and 5J and Figure 8–figure supplement 2B
PR672	*che-1(p672) I*	"ASE(-)" in Figures 1E,5J,6G
JN1715	*peIs1715 [str-1::mCasp-1, unc-122::mCherry]*	"AWB(-)" in Figure 1E,5J,7F
JN1713	*peIs1713 [sra-6::mCasp-1, unc-122::mCherry]*	"ASH(-)" in Figures 1E,5J
CF1903	*glp-1(e2141ts) III*	*"glp-1"* in Figure 5–figure supplement 5I
